# Health-Related Quality of Life in Children and Adolescents with Celiac Disease: From the Perspectives of Children and Parents

**DOI:** 10.1155/2012/986475

**Published:** 2012-04-03

**Authors:** Ing-Marie Byström, Elisabet Hollén, Karin Fälth-Magnusson, AnnaKarin Johansson

**Affiliations:** ^1^Division of Nursing Science, Department of Medical and Health Sciences, Faculty of Health Sciences, Linköping University, 581 85 Linköping, Sweden; ^2^Division of Medical Microbiology, Department of Clinical and Experimental Medicine, Faculty of Health Sciences, Linköping University, 581 85 Linköping, Sweden; ^3^Division of Pediatrics, Department of Clinical and Experimental Medicine, Faculty of Health Sciences, Linköping University, 581 85 Linköping, Sweden

## Abstract

*Aim*. To examine how celiac children and adolescents on gluten-free diet valued their health-related quality of life, and if age and severity of the disease at onset affected the children's self-valuation later in life. We also assessed the parents' valuation of their child's quality of life. *Methods*. The DISABKIDS Chronic generic measure, short versions for both children and parents, was used on 160 families with celiac disease. A paediatric gastroenterologist classified manifestations of the disease at onset retrospectively. *Results*. Age or sex did not influence the outcome. Children diagnosed before the age of five scored higher than children diagnosed later. Children diagnosed more than eight years ago scored higher than more recently diagnosed children, and children who had the classical symptoms of the disease at onset scored higher than those who had atypical symptoms or were asymptomatic. The parents valuated their children's quality of life as lower than the children did. *Conclusion*. Health-related quality of life in treated celiac children and adolescents was influenced by age at diagnosis, disease severity at onset, and years on gluten-free diet. The disagreement between child-parent valuations highlights the importance of letting the children themselves be heard about their perceived quality of life.

## 1. Introduction

Celiac disease (CD) is a persistent intolerance to gluten causing a mucosal damage of the small intestine in genetically susceptible individuals. It is one of the most common food-related chronic diseases and it often emerges during childhood. Genetic and environmental factors interact in the pathogenesis, and currently the only treatment is a life-long adherence to a strict gluten-free diet [1].

When a disease starts during childhood, the development, growth, self-concept, identity, and mental health of the child may be affected. Chronically ill children are more prone to physical, psychological, and social strains than healthy children, which may influence the child's health-related quality of life (HRQoL) in a negative way [2]. The problems are often more pronounced during school age, when it is pertinent for the child not to be deviant from other children [3, 4]. The gluten-free diet may cause problems for the child and its family. It can be hard for the child both to accept and comply with the strict diet. Alienation, shame, fear of eating something that contains gluten, and a feeling of being a nuisance are some of the factors related to CD [5]. A high incidence of psychological problems, for example, anxiety and depression, has been reported in CD children compliant to a strict gluten-free diet [6]. Relatives of CD patients often worry about how the person with CD will manage their everyday life and their social life [7]. Parents of children with chronic diseases often describe a large need of professional support, education, and guidance in questions concerning the child's disease [8].

There have been several studies concerning the HRQoL of adults with CD [5, 7, 9–12] but only a few studies comprise children [13–15]. In this study, we have examined how children and adolescents with CD valued their present HRQoL, and also if age, sex, and manifestation of the disease at onset affected the children's later valuation of their HRQoL. Furthermore, there are studies showing discrepancies between parents' and children's reports on the HRQoL [16, 17]; hence, we also compared the parents' valuation of their child's HRQoL with the corresponding assessment done by their child.

## 2. Patients and Methods

### 2.1. Patients

The study comprises children with treated CD and their parents, who visited the pediatric clinics in the south east of Sweden, that is, Linköping, Norrköping, Motala, and Västervik, for their annual follow-up in 2006-2007. A total of 160 families, with CD children 8–18 years of age, were asked to participate and all of them agreed. The children were administered the child's version of the questionnaire and the parents were asked to fill in the proxy version. The questionnaires were both filled in and handed in at the time of the visit at the clinic. Children with both CD and diabetes, with poor understanding of the Swedish language, or with cognitive difficulties were not included in the study. In nine of the participating families, there were two siblings with CD.

### 2.2. Measures

The study is a cross-sectional study with consecutive selection. The subjective health status of the children during the last four weeks was assessed using the Swedish version of the DISABKIDS Chronic generic measure (DCGM-12, short version) [18], a questionnaire where the child estimates its quality of life based on three domains: mental health, social health, and physical health. The questionnaire is constructed to address chronically ill children between 8 and 18 years of age. There is also a proxy version of the questionnaire, where the parents estimate the quality of life of their child. The DISABKIDS questionnaire is a well-validated test, *α* = 0.84 (the child version) and *α* = 0.86 (the proxy version) [18], and it is translated into several languages including Swedish [19]. The questionnaire is available in both a long and a short version, and the short version was used for this study.

The domain mental health contains four questions about independence, including autonomy and ability to live without restrictions due to the disease, and emotion, including anxiety, anger, and worries. The domain social health contains two questions concerning social community, including acceptance by and good relations to others, and two questions concerning social exclusion, including shame and feeling of exclusion. Two questions in the domain physical health concern functional limitations and subjective physical health status. In this domain, there are also questions concerning medical treatment, which are of no relevance for this study. A 5-graded Likert scale scores each question, where high scoring represents high HRQoL. At the analysis, each question was recoded from 1–5 points to 0–100 points, according to the user's manual for DISABKIDS.

### 2.3. Manifestation of Disease at Onset

The severity of the disease at onset was estimated retrospectively by an experienced paediatric gastroenterologist. The classification was done according to Fasano and Catassi [1], describing three groups of clinical presentations. Classical (typical) form means that the child had the typical celiac symptoms and signs at onset, that is, diarrhea, failure to thrive, loss of weight, great fatigue, enlarged abdomen, recurrent infections, and low serum albumin levels. Atypical form means a less pronounced onset, often with no typical gastrointestinal symptoms. Asymptomatic means that the child had no obvious symptoms and the investigation was prompted when a close relative got the diagnosis of CD. The health estimations were scored as follows: “Classical form” = 1 point, “Atypical form” = 2 points, and “Asymptomatic form” = 3 points.

## 3. Ethics and Statistics

Informed consent was received from all the participating parents, and the study was approved by the Research Ethics Committee at the Faculty of Health Sciences, Linköping University, Linköping, Sweden.

Since data were not normally distributed, nonparametric tests were used. For analysis of quantitative data (e.g., male/female), the Mann-Whitney *U*-test was used, and Wilcoxon's test was used for comparisons between paired groups (e.g., child/parents). When comparing three or more groups, the Kruskal-Wallis test was used. The relationship between variables was analyzed using Spearman correlation analysis. All analyses were performed using GraphPad Prism (version 5.0d for Mac OS X, GraphPad software, San Diego, CA), and *P* values equal or less than 0.05 were considered significant. If nothing else is indicated, all values are presented as median (25th percentile–75th percentile).

## 4. Results

The study comprises 160 children with confirmed celiac disease, 55 males and 105 females. Median age at inclusion, that is, when the questionnaire was filled in, was 13 years (range 8–18 years), and the median time since the diagnosis of CD was 10 years (range 1–17 years). The children were diagnosed between the years 1989 and 2006. A high percentage of the included cases (43%) were diagnosed between 1992 and 1996. The children were divided into three age groups: 8–11 years (*n* = 42), 12–15 years (*n* = 104), and 16–18 years (*n* = 14). The final response rate was 97.5% (*n* = 156, 54 males, 102 females) among the children and 95% (*n* = 152) among the parents. One child-questionnaire was ruined and three parents were visiting the clinic without their children, hence the loss among the children (*n* = 4). Eight adolescents visited the clinic without their parents, explaining the loss among the parents (*n* = 8). Hence, the results from comparisons between children and parents are presented from 149 child/parents pairs. Age and sex distribution, as well as the response rate, are shown in Table 1.

### 4.1. Total Score

The median value of the children's total score was 92 points (85.5–96). The median values in the separate specific domains were mental health 85 points (75–95), social health 95 points (85–100), and physical health 100 points (90–100).

### 4.2. Sex and Age

Sex and age did not correlate with the children's HRQoL score (*P* = 0.59 and *P* = 0.82, resp.). The only difference was seen in the age group 8–11 years, in which the boys (*n* = 16) scored lower than the girls (*n* = 25) in the domain physical health, with scores of 95 (90–100) and 100 (100-100), respectively (*P* = 0.05).

### 4.3. Years Since Diagnosis

The years since diagnosis were weakly (*r* = 0.26) but significantly (*P* < 0.001) correlated with the children's self-assessed quality of life. Those who received their diagnosis nine or more years ago valued their HRQoL higher than those who received it more recently (1–8 years), with scores of 92 (58–96) and 90 (36–94), respectively (*P* = 0.02). This was true also in the domain mental health where the groups scored with the median of 90 (55–95) and 85 (35–90), respectively (*P* = 0.01).

### 4.4. Age at Diagnosis

**Figure 1:**
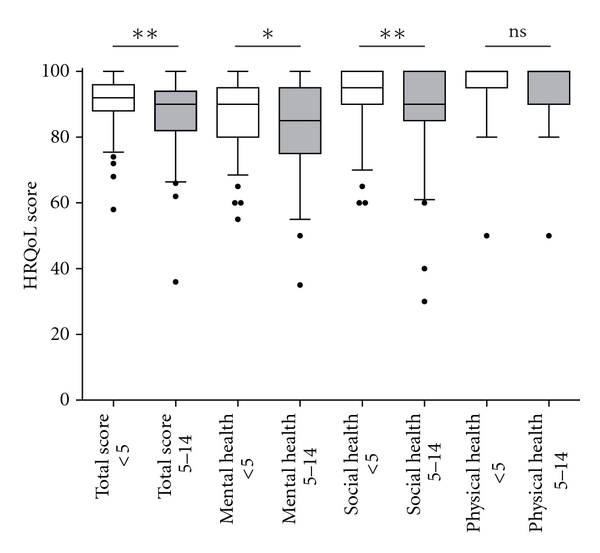
When assessing the health-related quality of life (HRQoL), using the DISABKIDS test, the children who received the diagnosis celiac disease before the age of five (*n* = 93) (<5, open boxes) scored higher in the total test, as well as in the domains mental and social health, as compared to children who were five years or older at the time of diagnosis (*n* = 63) (5–14, filled boxes). Box plot shows the median and 25th and 75th percentiles. Error bars represent 10th and 90th percentiles. **P < 0.01; *P < 0.05; ns: not significant.

Age at diagnosis was negatively correlated with the HRQoL score (*P* < 0.001). The children who received the diagnosis before the age of five (*n* = 93) scored higher than to those who were five years old or more (*n* = 63) at the diagnosis, with scores of 92 (88–96) and 90 (82–94), respectively (*P* = 0.006) (Figure 1).

In the domain mental health, the children diagnosed before or after five years of age scored with the median of 90 (55–95) and 85 (35–95), respectively (*P* = 0.03), and in the domain social health median 95 (90–100) and 90 (85–100), respectively (*P* = 0.01) (Figure 1).

### 4.5. Disease Manifestation at Onset

The children who presented with the classical form of CD (*n* = 68) valued their present HRQoL as higher than those who presented with atypical form (*n* = 74) or those who were asymptomatic (*n* = 14) with scores of 92 (88.5–97.5), 90 (84–94), and 88 (80.5–94.5), respectively, with a significant difference between the classical and the atypical group (*P* = 0.03). The median age in the three groups was 1, 7, and 7.5 years, respectively. The disease state at onset was found to be correlated with the children's total score (*P* < 0.05).

### 4.6. Parents/Children

**Figure 2:**
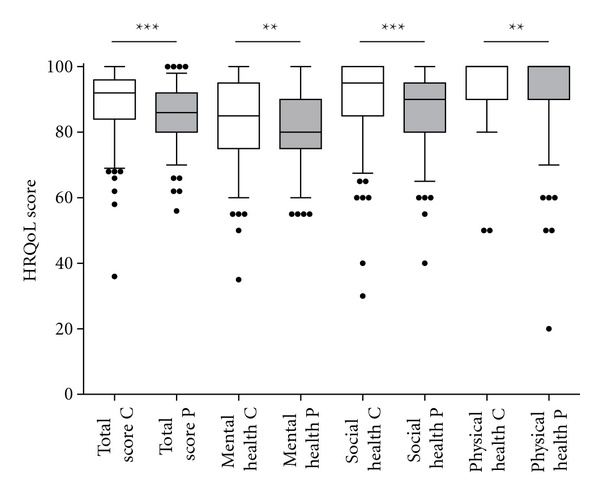
The parents (P, filled boxes) valued their children's health-related quality of life (HRQoL) as lower than the children themselves did (C, open boxes), both in the total score and in the three domains mental, social, and physical health. Box plot shows the median and 25th and 75th percentiles from 149 child/parents pairs. Error bars represent 10th and 90th percentiles. ***P < 0.001; **P < 0.01.

The parents' median total score for their children's HRQoL was 86 (80–92) while the children's median total score was 92 (84–96), and this difference was significant (*P* < 0.001), although the values were well correlated (*r* = 0.43, *P* < 0.001). The parental estimations were lower also in the specific domains: in mental health the parents' and the children's median score was 80 (75–90) and 85 (75–95) (*P* = 0.003); in social health 90 (80–95), and 95 (85–100) (*P* < 0.001); and in the domain physical health 100 (20–100) and 100 (50–100) (*P* = 0.005), respectively (Figure 2).

Age and sex of the children did not correlate with the parents' valuation of the children's HRQoL, neither did the manifestation of the disease at onset. However, there were significant differences in the estimation of the HRQoL if the child was diagnosed before or after the age of five (median 86, 82–94 and median 84, 78–90, resp.) (*P* = 0.02). There was also a weak, but significant, correlation between the parents' estimation and the duration of the disease for the child (*r* = 0.18, *P* = 0.03).

## 5. Discussion

In this study, we have assessed the subjective health-related quality of life in children and adolescents with treated CD, and the corresponding evaluation made by their parents. We found that the children valued their quality of life as very high, but the parents estimated their children's HRQoL as lower than their children.

The results suggest that the children, as a group, have adapted well to the disease. Indeed, in two earlier studies [14, 15], the authors claim that children with CD who keep a strict gluten-free diet experience the same high HRQoL as healthy children. While being aware that we could lower the sensitivity and specificity by not choosing a disease specific measure [20], we have used the DISABKIDS chronic generic measure, which is a well-established and validated instrument for assessing HRQoL in children with chronic diseases [18]. The instrument may be used for different diagnostic groups, and this makes comparisons possible on how distinct diseases affect children's self-estimated quality of life. In a pilot study done by the DISABKIDS group, other chronic diseases, such as asthma, arthritis, dermatitis, diabetes, cerebral palsy, cystic fibrosis, and epilepsy, were tested [18]. The mean total score for these conditions ranged between 63 and 81 points, with the cerebral palsy children scoring lowest and the asthma children highest. There were no reference values for celiac disease; hence, the present study is to best of our knowledge the first to show values for CD using this instrument. The CD children in our study scored higher than the children affected by other chronic diseases both in total score and in the three domains of the questionnaire.

Measurements of HRQoL based on life situations are difficult to perform, since both subjective and objective circumstances must be taken into consideration. It has been reported that children's compliance to a gluten-free diet was correlated with the parent's knowledge and understanding of the disease. This in turn was highly correlated with the social status of the families [21]. In the present study, neither the socioeconomic factors of the family, nor the parents' occupation, education, or the living environment have been highlighted. However, in order to elucidate the significance of different factors on the quality of life, we related the HRQoL of the children to age, sex, years since diagnosis, age at diagnosis, and the severity of the disease at onset. The results show that sex had no impact on the HRQoL, neither had age as a single factor.

Children who were diagnosed before the age of five scored better than those who were five years or older at the diagnosis. Furthermore, we noted that children who had had their disease for a long time experienced their current quality of life as higher. These results are consistent with the report from Högberg et al. [22], where children who were diagnosed before the age of four accepted their illness and the gluten-free diet better than those who were diagnosed later. The young children have probably not been accustomed to the taste of gluten-containing food, which may result in a better compliance to the gluten-free diet. There are studies showing that it may be difficult to adapt to a chronic disease during adolescence, the period of life when the needs to be like the others probably are the highest [3, 23, 24].

An additional factor influencing the children's HRQoL was the manifestation of the disease at onset. The classification into three groups was done retrospectively by an experienced paediatric gastroenterologist, thereby increasing the reliability of the assessment. The children who presented with the classical form of the disease valued their HRQoL as highest. The majority of the children in this study who were diagnosed before the age of four presented with the typical CD symptoms, for example, diarrhea, failure to thrive, loss of weight, and enlarged abdomen. Currently, the median age at diagnosis has increased, and children receive their diagnosis more often at an older age and present with more diffuse symptoms [25]. Further studies should be performed on the latter patient group, using the same instrument, in order to reveal the importance of age at diagnosis and disease severity as predictors of HRQoL later in life.

 Some children in this study had extremely low scores in HRQoL (Figure 2). We cannot explain if this is due to celiac disease or due to other factors that were not assessed in this study. Yet, such low values should alert the health professionals to evaluate the need for psychological and social support.

According to Eiser and Morse [26], parents with chronically ill children are able to make a better valuation of their child's HRQoL as compared to parents with healthy children. In the present study, the parents valued their children's HRQoL as lower than the children themselves did. This was noted both for the total quality of life and for the three domains mental, social, and physical health. Sawyer et al. [27] described how health care professionals often listen only to the parent's description of the child's problems, which may lead to a misinterpretation of the child's HRQoL by the staff and a risk for overtreatment of the child. Parents of CD children are often worried about possible complications, development of other autoimmune diseases, fertility, and heredity. They are also concerned about what the children have to endure, and what they have to abstain from, and indeed, the greatest discrepancy between children and parents in this study was seen in the domain social health. The parent's lower valuation of their children's HRQoL may also be due to the parent's sense of responsibility and concern as the basis for the valuation [27]. Many parents feel guilt, sadness, bitterness, and difficulties of coping in the everyday life when their child is diagnosed with a chronic disease. Indeed, parents in all the diagnostic groups in the pilot studies in DISABKIDS valued the children's HRQoL lower than their children did [18]. Interestingly, when assessing HRQoL in the parents of children with CD, de Lorenzo et al. [13] found an impaired self-valuation in comparison to parents of healthy children, especially in the social dimension. This suggests an impact of the diet regimen and possibly other factors on the parents and on close relatives, a suggestion that was also proposed by Sverker et al. [7].

The parent's valuation was also affected by the age of the child at the time of diagnosis. The younger the child was at onset, the better was the parent's valuation of its present HRQoL. This may be due to the fact that the youngest children also had the most severe symptoms of the disease and, hence, were the ones that most obviously benefitted from the gluten-free diet. Furthermore, parents of children who had the diagnosis for a long time tended to value their child's quality of life higher, possibly reflecting that the families were getting used to the diet regimens and all the difficulties that could come out of it.

One important limitation of the present study was the lack of a control group of healthy individuals. However, the instrument used in the study was developed to address children with chronic diseases making the use of a healthy control group difficult. Furthermore, while our study group had higher HRQoL than groups with other chronic diseases, other studies reported that children with CD generally had high values in HRQoL measurements and that the values were similar to the control groups [13, 14]. On the contrary, when assessing psychological symptoms in treated CD children and controls, Mazzone et al. [6] found signs of more depression and anxiety in the CD group, indicating an influence of the strict diet regimen on the child's psychological well-being, something that health care professionals should be aware of.

The reason for using HRQoL instruments in health care is to get a combined picture of the mental, social, and physical health of the child. This could help the health care professionals in getting a clearer view of how the children and their relatives experience chronic diseases. In the present study, it was important that both the celiac child and the parents made an estimation of the child's current HRQoL, in order to improve our knowledge of the living conditions for celiac families.

In conclusion, children who were diagnosed before the age of five and who presented with the classical form of CD scored their health-related quality of life higher. The children who have had the disease for a longer time also scored higher. The celiac children in this study scored higher than other diagnostic groups assessed with the same instrument. Notably, the parents scored significantly lower than the children when they were asked to evaluate their child's HRQoL. The disagreement between the self- and the proxy valuations highlights the importance of letting the children themselves be heard about their perceived quality of life.

## Figures and Tables

**Table 1:** Age and sex distribution of the study population and the response rate of the questionnaires.

Number of participating families				Response rate, *n*			
Age	Total	Female	Male	Children	Female	Male	Parents
8–11	42	25	17	41	25	16	42
12–15	104	67	37	102	65	37	100
16–18	14	13	1	13	12	1	10

Total	160	105	55	156^a^	102	54	152^b^

^a^One questionnaire was broken and three parents came to the clinic without their children.

^b^Eight adolescents visited the clinic without their parents.

## Abbreviations

CD: Celiac disease
HRQoL: Health related quality of life.
